# Case report: Severe hypertension-induced priapism in an infant with unrecognized autosomal recessive polycystic kidney disease

**DOI:** 10.3389/fped.2023.1216239

**Published:** 2023-09-12

**Authors:** Patrik Konopásek, Natálie Ptáčníková, Ledjona Toni, Jakub Zieg

**Affiliations:** ^1^ Department of Pediatric Nephrology, 2nd Faculty of Medicine, University Hospital Motol, Charles University, Prague, Czechia; ^2^ Department of Biology and Medical Genetics, 2nd Faculty of Medicine, Charles University and Motol University Hospital, Prague, Czechia

**Keywords:** priapism, hypertension, infant, autosomal recessive polycystic kidney disease, sickle cell disease

## Abstract

Priapism is a urologic emergency requiring prompt management. There are three types of priapism: stuttering (intermittent), non-ischemic (high-flow/arterial), and ischemic (low-flow/veno-occlusive). Here, we present the first case of an infant with recurrent non-ischemic priapism as the first sign of severe hypertension. An 11-month-old infant was admitted to the hospital for high-flow priapism. On admission, he was found to have severe hypertension that required a combination of five antihypertensive drugs; abdominal ultrasound showed polycystic kidneys, splenomegaly, and a parenchymal liver lesion. The priapism resolved spontaneously and did not recur again after the initiation of antihypertensive treatment. Genetic analysis confirmed autosomal recessive polycystic kidney disease (ARPKD). We found no other explanation for the priapism, such as genital trauma, hematologic disease, or anything else. Decreased nitric oxide (NO) bioavailability seen in patients with hypertension seems to be the principal mechanism of hypertension causing priapism. This hypothesis is supported by animal models of genetically modified mice lacking nitric oxide synthase. The same mechanism is thought to be the genesis of priapism and other complications, such as pulmonary hypertension, in patients with sickle cell disease. We present a case of severe hypertension-associated priapism in a child with unrecognized ARPKD. The endothelial dysfunction with decreased NO bioavailability seen in patients with hypertension may be the principal pathogenic mechanism.

## Introduction

Priapism is defined as a penile erection that lasts for more than 4 h without sexual stimulus. It is traditionally classified as ischemic (low-flow), stuttering (recurrent ischemic), or non-ischemic (high-flow). While ischemic priapism should be treated promptly, the diagnosis of high-flow priapism made by Doppler ultrasound does not require urgent treatment. Hematologic diseases, such as leukemia and thalassemia, are the principal causes of ischemic priapism, with sickle cell disease (SCD) being the most frequent in both ischemic and stuttering priapism (1). The classical theory says that increased blood viscosity leads to congestion and slower blood flow, causing ischemia, which is the main cause of low-flow priapism (2). Non-ischemic priapism results from genital trauma and the creation of a fistula with high blood flow (3). We present a case of a child with unrecognized autosomal recessive polycystic kidney disease (ARPKD) who presented with priapism, probably due to severe uncontrolled hypertension (HT).

## Case report

**Figure 1:**
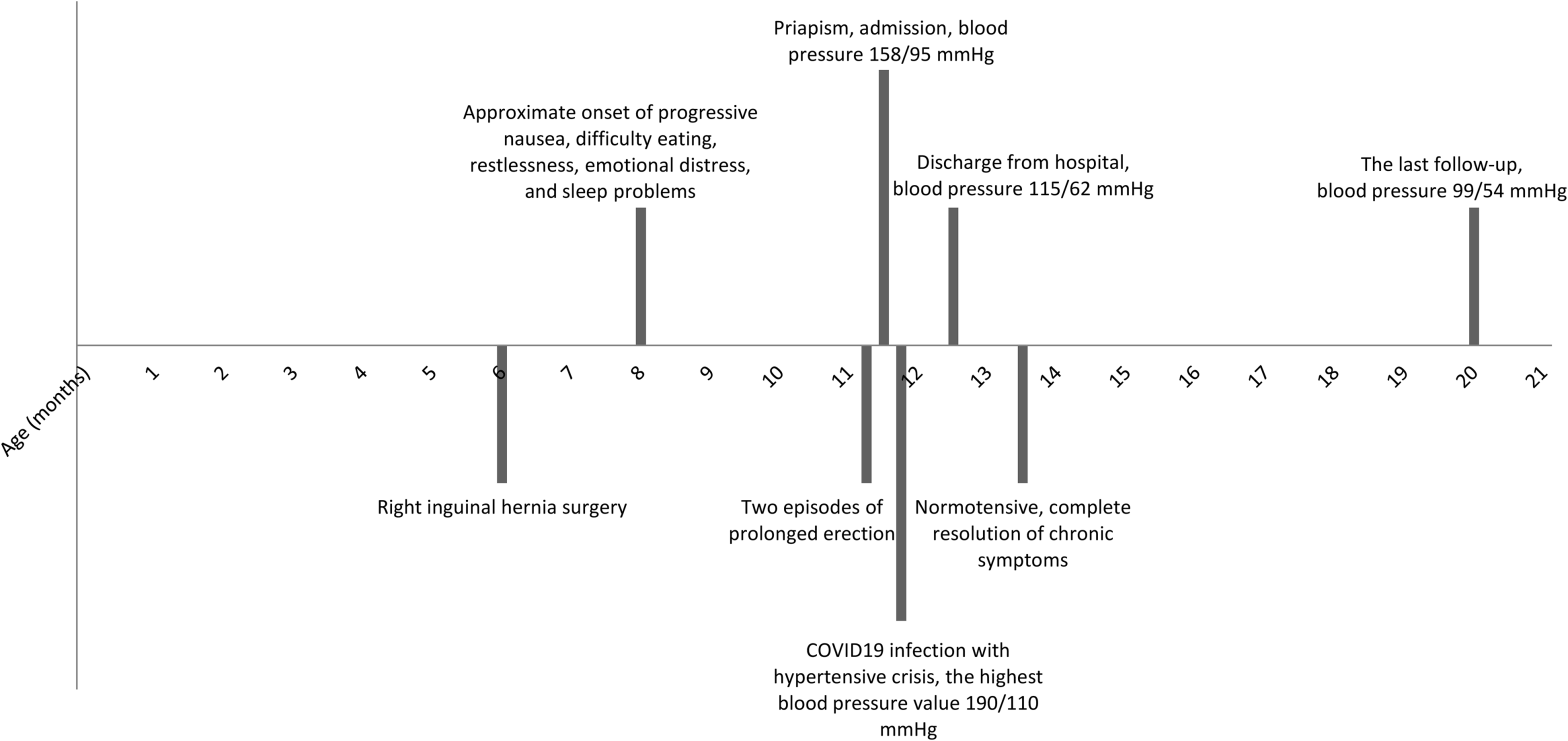
Graphical timeline.

An 11-month-old infant was transferred to our pediatric nephrology unit with a 30-h-long history of non-painful priapism. During the week before the first examination, the parents noticed two episodes of penile erection lasting approximately 2 h, which had never previously occurred for such a long period of time. From the patient's medical history, prenatal ultrasound was performed during the first, second, and third trimesters of pregnancy with no pathology described. The little boy underwent regular physiotherapy for axial hypotonia and had right inguinal hernia surgery performed at the age of 6 months. His parents noticed progressive nausea, feeding difficulties, restlessness, emotional distress, and sleeping problems every night for the past few months (Figure 1). His other medical history was unremarkable, but his blood pressure (BP) had never been measured. Physical examination revealed an infant whose weight and height were 8,680 g and 74 cm, respectively, with psychomotor development retardation (PDR), severe HT (158/95 mmHg), being the first recorded blood pressure, and priapism without visible ischemia. Abdominal and penile ultrasounds (US) were performed immediately after admission to quickly assess the type of priapism. The radiologist described high-flow priapism on Doppler US of the penis, renomegaly (right kidney length 88 mm, left kidney length 84 mm), polycystic kidneys, mild splenomegaly, and a parenchymal liver lesion (hyperechogenic liver with periportal hypoechogenic structures without ductal dilatation) on the abdominal US. The priapism resolved spontaneously within 1 h. The corporal aspiration was not indicated by the pediatric surgeon for two reasons: First, the Doppler US showed a non-ischemic type of priapism, and, more importantly, this resolved spontaneously when the surgeon was present. We evaluated the possible risk factors for arteriovenous fistula (AVF), but there was no relevant medical history or clinical signs of genital or pelvic trauma. Blood analyses showed thrombocytopenia, elevated plasma renin activity, and mild elevation of liver transaminases without any other pathology; urine analyses showed no hematuria or proteinuria (Table 1). Echocardiography revealed left ventricular hypertrophy (left ventricular mass index (LVMI) = 111.2 g/m^2.7^) with normal function, and eye fundoscopy showed no pathology. Hemoglobin electrophoresis was normal. As ARPKD was our initial likely diagnosis, molecular genetic testing using a next-generation sequencing-based panel of 486 genes associated with genetic kidney disease was performed, along with evaluation for inherited metabolic diseases (IMDs) because PDR was also present. An MRI of the central nervous system (CNS) was performed, showing no pathology. We considered HT to be chronic due to suspected ARPKD and the presence of target organ damage, and oral antihypertensive treatment was commenced with gradual dose increases. The course was then complicated by a hypertensive crisis during an acute COVID-19 infection, which was well controlled with oral therapy. Normal BP values were then achieved with a combination of five oral drugs (captopril, carvedilol, hydrochlorothiazide, amlodipine, and off-label urapidil) 1 month after admission. The patient’s BP was 99/54 mmHg at the last follow-up. Adequate compensation of HT led to the resolution of sleeping problems and nausea. There have been no further episodes of priapism since the start of the antihypertensive treatment. Molecular genetic analysis confirmed compound heterozygous pathogenic variants in the *PKHD1* gene (NM_138694.4): c.107C >T (p.Thr36Met) and c.474G >A (p.Trp158*). The variants are in *trans*, confirming the diagnosis of ARPKD. No IMDs were found. We conclude that severe unrecognized HT in our patient with ARPKD caused PDR, nausea, and priapism.

**Table 1:** Basic blood and urine analysis.

Parameter	Value (reference values)	Parameter	Value (reference values)
Hemoglobin (g/L)	126 (105–135)	Albumin (g/L)	43.1
Platelets (×10^9^/L)	105 (150–450)	Total protein (g/L)	58
Leukocytes (×10^9^/L)	8.3 (6.0–17.5)	Uric acid (μmol/L)	385
Sodium (mmol/L)	137	Parathormone (pmol/L)	0.85
Potassium (mmol/L)	5	TSH (mIU/L)	2.648
Chloride (mmol/L)	108	fT4 (pmol/L)	19.84
Calcium (mmol/L)	2.57	Aldosterone (nmol/L)	0.419 (0.72–39.6)
Magnesium (mmol/L)	0.86	PRA (ng/ml/hod)	19.94 (0.32–1.84)
Phosphate (mmol/L)	1.55	Metanephrine (nmol/L)	0.17 (0.1–0.5)
AST (µkat/L)	1.4 (0.27–0.97)	Normetanephrine (nmol/L)	0.9 (0.1–0.9)
ALT (µkat/L)	0.9 (0.15–0.85)	U-erythrocytes	0 cells
GGT (µkat/L)	0.75 (0.1–1.04)	U-protein/creatinine ratio	Not measurable
Urea (mmol/L)	5.1	U-albumin/creatinine ratio	Not measurable
Creatinine (μmol/L)	26 (4–35)	GFR (Schwartz equation)	104 ml/min/1.73 m^2^
Glycemia (mmol/L)	5.2	LD (μkat/L)	5.3 (3–7.16)
CK (μkat/L)	2.06 (0.17–2.44)	Cholinesterase (μkat/L)	157 (116–316)
Amylase (μkat/L)	0.40 (0.3–2.18)	Total bilirubin (μmol/L)	5.2 (5–21)
Cortisol (nmol/L)	268.2	Ferritin (μg/L)	12.4
Cholesterol (mmol/L)	4.3	HDL (mmol/L)	1.01
LDL (mmol/L)	3.17	Alpha-fetoprotein (μg/L)	11.65 (0–77)
25D (nmol/L)	148.8	aPTT (s)	30
INR	0.95	Fibrinogen (g/L)	2.20
Antithrombin III (%)	119	Parathormone (pmol/L)	0.85

25D, 25-hydroxycholecalciferol; ALT, alanine aminotransferase; aPTT, activated partial thromboplastin time; AST, aspartate aminotransferase; CK, creatine kinase; fT4, free thyroxine; GFR, glomerular filtration rate; GGT, gamma-glutamyl transferase; HDL, high-density lipoprotein cholesterol; LD, lactate dehydrogenase; LDL, low-density lipoprotein cholesterol; INR, international normalized ratio; PRA, plasma renin activity; TSH, thyroid-stimulating hormone; U, urine.

## Discussion

ARPKD is a severe genetic disorder characterized by the formation of kidney cysts and defects in the hepatobiliary ductal plate remodeling, leading to progressive hepatopathy and chronic kidney disease (CKD). Perinatal mortality is high, mainly due to pulmonary hypoplasia. Fetal sonography performed at the time when pregnancies are usually terminated may fail to detect abnormalities normally seen in patients with ARPKD; thus, early and reliable prenatal diagnosis of ARPKD in at-risk families is feasible only by molecular genetic analysis. HT is very common in patients with ARPKD who have presented with this condition since early childhood and may be difficult to control (4).

To the best of our knowledge, no association between HT and priapism has been reported to date. Normally, limited cavernosal arterial inflow caused by high resting arterial and cavernosal smooth muscle tone causes penile flaccidity. The production of nitric oxide (NO) catalyzed by endothelial nitric oxide synthase (NOS) plays a critical role in erection by relaxing the smooth muscle, which leads to an increase in arterial inflow (1). On the other hand, chronically impaired NO bioavailability is also associated with priapism (5). This condition may be seen in patients with SCD where hemolysis leads to endothelial dysfunction with subsequent reduced NO bioavailability and increased NO resistance, which is thought to be the genesis for priapism and other SCD complications, such as pulmonary HT (6). Consistent with this, genetically modified mice lacking NOS have been shown to have increased priapic activity, pronounced erectile responses, and prolonged erections after discontinuation of the stimulus (7).

Under normal circumstances, endothelial cells regulate the vascular tone through the synthesis of NO, prostaglandins, and other relaxing factors. In patients with HT, a reduction of NOS occurs, resulting in decreased production and bioavailability of NO (8). Chronically impaired NO bioavailability due to unrecognized severe HT explains the possible mechanism of priapism in our patient. AVF is the most common etiology of high-flow priapism; however, we did not detect any possible risk factors for AVF formation. We found no other explanation for the priapism other than unrecognized severe HT, and no further episodes of priapism have occurred since the antihypertensive treatment was initiated. The question is why there have not been previous reports on the association between HT and priapism. One of the reasons could be the severity of the unrecognized chronic HT in our patient, who was not able to complain of any symptoms, while another could be that, in the case of priapism, the possible association with HT is not considered.

In conclusion, we present the first case of priapism associated with unrecognized severe HT in an infant with ARPKD. Decreased NO bioavailability, as described in patients with HT, seems to be the principal mechanism.

## Data Availability

The original contributions presented in the study are included in the article; further inquiries can be directed to the corresponding author.
